# Factors Influencing the Aged in the Use of Mobile Healthcare Applications: An Empirical Study in China

**DOI:** 10.3390/healthcare11030396

**Published:** 2023-01-30

**Authors:** Xiang Wang, Chang-Franw Lee, Jiabei Jiang, Xiaoyang Zhu

**Affiliations:** 1 Graduate School of Design, National Yunlin University of Science and Technology, Yunlin 64002, Taiwan; 2 Pujiang Institute, Nanjing Tech University, Nanjing 211200, China; 3 The Future Laboratory, Tsinghua University, Beijing 100080, China; 4 School of Arts and Design, Sanming University, Sanming 365004, China

**Keywords:** the aged, mobile healthcare application, technology acceptance model, protection motivation theory, perceived risk theory, structural equation model

## Abstract

Mobile healthcare applications are of significant potential value in the development of the aged-care industry due to their great convenience, high efficiency, and low cost. Since the cognition and utilization rates of mobile healthcare applications for the elderly are still low, this study explored the factors that affect the elderly’s adoption of mobile healthcare applications. This study conducted a questionnaire survey on the elderly in China and received 365 valuable responses. This study combined the technology acceptance model, protection motivation theory, and perceived risk theory to build a research model of factors affecting the use of mobile healthcare applications by the elderly. The data were analyzed using a structural equation model. The results were as follows: according to the empirical research, (1) perceived usefulness and perceived ease of use positively affect the use attitude of the elderly; perceived usefulness and user attitude positively affect the behavior intention of the elderly; perceived ease of use positively affects perceived usefulness; (2) perceived severity has a significant positive correlation with use attitude; perceived susceptibility and attitude to use have no significant impact; (3) perceived risk is negatively correlated with the use attitude and behavioral intention. The above-mentioned factors should be taken into consideration during the development of mobile healthcare applications for the aged to upgrade the overall service quality of mobile healthcare applications, thus enhancing the operational level of mobile healthcare applications and the health literacy of the aged.

## 1. Introduction

### 1.1. Background

According to the seventh China Census, there are 264.02 million people aged 60 and above, accounting for 18.7% of the total population in China, and 190.64 million people aged 65 and above, accounting for 13.50%. It thus appears that China is faced with a serious problem of population aging [1]. The health of the aged has gradually become the focus of public attention as the aging problem is turning into an increasingly severe issue. In 2015, UN member states put forward the 2030 Agenda for Sustainable Development [2], of which Goal 3 was explicitly stipulated as “Good health and well-being”. With the enhancement of health consciousness among the aged, there are growing medical and health demands among the aged. The rise and progress of mobile health provide new insights into the management of physical conditions of the aged. Featuring convenience, accessibility, high efficiency, and a low cost, this model is also consistent with the growing trend of informationization in current society [3,4,5]. On 11 March 2016, the General Office of the State Council issued the *Guiding Opinions of The General Office of the State Council on Promoting the Healthy Development of the Pharmaceutical Industry* to encourage the growth of mobile health and also put forward the requirements for the development of health-related applications [6].

Mobile healthcare (mHealthcare) refers to the use of mobile devices (e.g., smartphones, patient testing equipment, and personal digital products) to facilitate medical treatment and health management. mHealthcare has various forms, including both short-term services such as SMS reminders for smoking cessation and weight loss, and long-term services for disease cure, for instance, the monitoring and control of illnesses such as diabetes and high blood pressure [7]. In the smartphone era, mHealthcare achieves physical condition monitoring and disease treatment control via a smart app using the feats of sensor technology, GPS, Bluetooth, and other technical forms [8]. Mobile healthcare applications are a type of technological innovation [9], with their significance and advantage lying in convenient mHealthcare medical advice and management services whenever and wherever possible, free from geographical, time, and even organizational barriers, and greatly facilitating the elderly and the health needs of the elderly and alleviating the social and economic pressure of the country [10].

### 1.2. Research Purpose

Many scholars have focused on research in the field of mobile health. In terms of contemporary research, on the one hand, there are relatively few studies on mobile healthcare applications in China with the elderly as the established population. On the other hand, previous studies on mobile health have mostly focused on the exploration of some healthcare technologies or the design, development, and application of mHealthcare applications and their effects [11,12,13,14,15,16], with little research focusing on user behavior.

In view of the current research situation and research gaps, this study focuses on the behavior of mobile healthcare application users among the elderly. Based on the literature, this study constructs a research model of the factors that affect the use of mobile healthcare applications by the elderly, aiming to explore the factors that affect the use of mobile healthcare applications from the perspective of technology, health behavior, and risk perception. This study obtains valuable conclusions through empirical research and analysis and provides effective suggestions for improving the overall service quality of mobile healthcare applications, the willingness of the elderly to adopt mobile healthcare applications, and the sustainable development of the mobile healthcare application industry.

## 2. Literature Research

As mobile healthcare applications are a relatively new technology information product for the elderly, the technology acceptance model can be used to explore the key factors affecting the use of mobile healthcare application technology by the elderly. As an activity to promote, protect, and maintain health, it is not sufficient to only analyze mHealthcare app adoption behavior from the perspective of technology, as it should also be studied from the perspective of health behavior. Therefore, the theory of protection motivation can be used to explore the key factors that influence the health of the elderly through the use of mobile healthcare applications. Perceived risk is due to the existence of uncertainty. The elderly often face many uncertain factors in the process of adopting mobile healthcare applications. This study will use the perceived risk model to explore the impact of perceived risk on the mobile healthcare application use of the elderly. Therefore, this paper integrates the technology acceptance model, protection motivation theory, and perceived risk theory into a new research model to analyze the behavioral willingness of the elderly to use mobile health services for health management.

### 2.1. Technology Acceptance Model (TAM)

**Figure 1:**
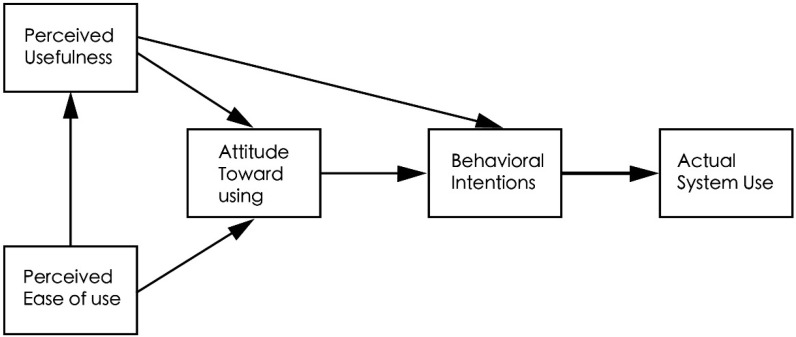
Technology acceptance model.

The technology acceptance model was proposed by Davis (1989) [17], aiming to study users’ acceptance of information technology, as shown in Figure 1. As indicated by this model, the perceived usefulness and the perceived ease of use jointly influence the users’ attitude, behavioral intention, and actual use of certain technologies. Perceived usefulness refers to an individual’s perception that a system or technology is beneficial. Perceived ease of use refers to an individual’s perception of how easy it is to operate or use a system or technology. Perceived ease of use will affect the perceived usefulness, both of which are further influenced by external variables such as design features and the content of systems and technologies.

Researchers believe that the TAM model features high forecasting validity in user technology acceptance, which leads to extensive use in various backgrounds concerning user technology acceptance [18,19,20,21,22]. Moreover, it has been proven that the model can effectively predict and explain users’ acceptance and adoption behaviors toward information systems [23]. As mHealthcare applications are a type of information system, the existing TAM research model and empirical method can be applied to this study.

### 2.2. Protection Motivation Theory (PMT)

Protection motivation theory was proposed by Rogers based on the Health Belief Model (HBM) [24], holding the view that, for better explaining the transformation of health-related behaviors, the factors affecting health behaviors mainly include severity, susceptibility, self-efficacy, and response efficacy [25]. On the basis of the threat assessment (severity, susceptibility) and coping assessment (self-efficacy, response effectiveness) during the perception process, protection motivation theory would determine whether to produce protection motivation, and eventually generate protection behaviors according to the comprehensive assessment [26]. PMT has been widely adopted by scholars in many studies, including chronic health education, AIDS prevention, hepatitis B, and safe sex, to explain, predict, and intervene in healthy behaviors [27]. Anderson and Agarwal [28] studied PMT and its relationship with attitude and intention, and found that threat assessment and coping assessment could significantly affect the adoption and behavioral intention of users of e-health and mHealthcare services. Some researchers have found that the influencing factors of PMT would firstly affect people’s attitude, and then their use behavior. The higher the threat assessment, the stronger the intention of behavior change, and the more capable the individual is to cope with the threat assessment, the easier it will be to change their behaviors [29].

PMT has been widely used to study the changes in users’ health behaviors [30]. In this study, mobile healthcare applications concern the health and safety of the aged, and feature the functions of providing health information, medical services, and health management. Accordingly, this study suggests that the aged will adopt mobile healthcare applications according to their own cognition of their health status.

### 2.3. Perceived Risk Theory

Perceived risk was first proposed in the field of psychology by Bauer (1960) [31] of Harvard University. According to this concept, individuals may experience a variety of unpleasant factors including personal loss, and the outcomes caused by their behaviors might be good or bad. People will predict the risk of behavioral outcomes before the initiation of such behavior, which is known as perceived risk. As for the consumer analysis in the market environment, Bauer stated briefly that perceived risk focuses more on the risks that the user subjectively feels, instead of the real individual behavior risk that is posed.

The theory of perceived risk is widely used in the field of mobile healthcare applications. For example, Klaver et al. [32] used perceived risk to discuss the application of mHealthcare for the elderly in the Netherlands. Sinha et al. [33] combined the TAM with perceived risk to explore the factors affecting consumers’ adoption of health information technology. Laili and Wahyudi [34] discussed the impact of the perceived risk of COVID-19 on the transaction intention of mHealthcare applications.

On the basis of former relevant studies [32,33,34,35,36,37,38], this paper defines perceived risk as the degree of prediction and loss acceptance of the aged to the risks caused by the use of mobile healthcare applications in their subjective consciousness.

## 3. Model Building and Hypotheses

### 3.1. Research Hypotheses

#### 3.1.1. Relation among Perceived Usefulness, Attitude toward Using Mobile Healthcare Applications, and Behavioral Intention

According to Davis, perceived usefulness is defined as the degree to which an individual believes that the use of a certain system will improve his/her work performance. It is a decisive factor affecting the acceptance and adoption of information technology by users and has an immediate crucial influence on their behavioral intentions. Generally speaking, the more performance an information technology system can bring to users, the more inclined users will be to adopt the system. Pan [39], Jiang et al. [40], Yu et al. [41], and other scholars’ empirical studies in different fields based on the TAM all assume that perceived usefulness is one of the variables determining behavioral intention. Empirical studies show that the higher the degree of perceived usefulness of an application becomes, the stronger the users’ behavioral intentions will be. The variable in this paper is defined as the extent to which the aged can access healthcare services and improve their health when using mHealthcare applications. Therefore, the following hypotheses are proposed in this study: Hypothesis 1, perceived usefulness is positively correlated with usage attitude; Hypothesis 2, perceived usefulness is positively correlated with behavioral intention. 

#### 3.1.2. Relation between Perceived Ease of Use and Attitude toward Using Mobile Healthcare Applications

Perceived ease of use is considered as the user’s perception of the simplicity, convenience, and ease of use of an information system. According to the theoretical thought of the TAM, if the technology is perceived as easy to understand and use, users will feel interested and use the system more actively with a stronger intention of acceptance. The same research conclusions have been reached by Luo and Zhu [42], Zhu and Guo [19], Li and Zhang [20], and other scholars: perceived ease of use will have a direct or indirect positive impact on users’ behavioral intention. In this study, perceived ease of use in the context of mHealthcare applications is defined as the degree of difficulty that the aged feel during application use or learning. Therefore, we put forward Hypothesis 3: perceived ease of use is positively correlated with the attitude toward using mobile healthcare applications.

#### 3.1.3. Relation between Perceived Ease of Use and Perceived Usefulness

TAM theory acknowledges that perceived ease of use has a positive influence on perceived usefulness. Users’ perceived ease of use of the system does not always directly affect their behavioral intentions, largely because the ease of use of the system has relieved their antipathy toward the system, thus generating the perception of usefulness. Empirical studies [43,44] have proven that perceived ease of use is positively correlated with perceived usefulness. Therefore, we put forward Hypothesis 4: perceived ease of use is positively correlated with perceived usefulness.

#### 3.1.4. Relation between Attitude toward Using Mobile Healthcare Applications and Behavioral Intention

As indicated by the TAM, users’ acceptance behavior toward an information system is directly determined by their subjective intention of behavior, which is jointly determined by users’ attitude and perception of the information system. Empirical evidence shows that the attitude toward using mobile healthcare applications has a positive correlation with the behavioral intention [45,46]. Therefore, we put forward Hypothesis 5: attitude toward using mobile healthcare applications is positively correlated with behavioral intention.

#### 3.1.5. Relation among Perceived Susceptibility, Perceived Severity, and Attitude toward Using Mobile Healthcare Applications

Threat assessment mainly includes two influencing factors, namely, susceptibility and severity. Severity mainly refers to an individual’s judgment on the harm degree of a behavior to their own physical and mental health. Susceptibility mainly refers to the major belief formed by an individual’s subjective judgment about the possibility of suffering from a disease. Coping assessment mainly includes individual self-efficacy and response efficacy. Self-efficacy refers to an individual’s intuition of their own ability to take a certain protective behavior, that is, one’s prior belief, judgment, and subjective self-perception of their own ability in behavior fulfillment at a given level. Response efficacy refers to an individual’s perception of whether a certain protective behavior taken is effective. The more convinced one is of benefitting from the behavior taken, the stronger the motivation for that behavior [47]. Due to the similarity between the concepts of self-efficacy and response efficacy in PMT, and that of perceived ease of use and perceived usefulness in the TAM, this study only discusses the influence of threat assessment (susceptibility and severity) factors on the attitude toward using mobile healthcare applications. Based on previous studies [30,48,49,50,51], we put forward the following hypotheses: Hypothesis 6, perceived susceptibility is positively correlated with attitude toward using mobile healthcare applications; Hypothesis 7, perceived severity is positively correlated with attitude toward using mobile healthcare applications.

#### 3.1.6. Relation between Perceived Risk and Attitude toward Using Mobile Healthcare Applications

Taylor (1974) [52] held the view that users’ decision of adoption is concerned with their perceived risks. When the perceived risks are high, users are likely to form a negative attitude, thus diminishing their behavioral intention. Schnall et al. [53] believe that mobile health technology will cause consumer privacy and security concerns, and risk perception will significantly affect the technology adoption of mobile healthcare applications. Said [54] believes that mobile healthcare applications have brought many risks to Egyptian users, and the impact of perceived risks on technology adoption is significant. Zhao and Zhou [55] believe that perceived risk plays a significant role in the adoption of mobile healthcare applications. In this study, the perceived risk model is employed to elaborate on the impact of perceived risk on the attitude of the aged toward using mobile healthcare applications. Based on previous studies [35,36,37,38,53,54,55] and we put forward the following hypotheses: Hypothesis 8, perceived risk is negatively correlated with attitude toward using mobile healthcare applications; Hypothesis 9, perceived risk is negatively correlated with behavioral intention.

### 3.2. Model Building

**Figure 2:**
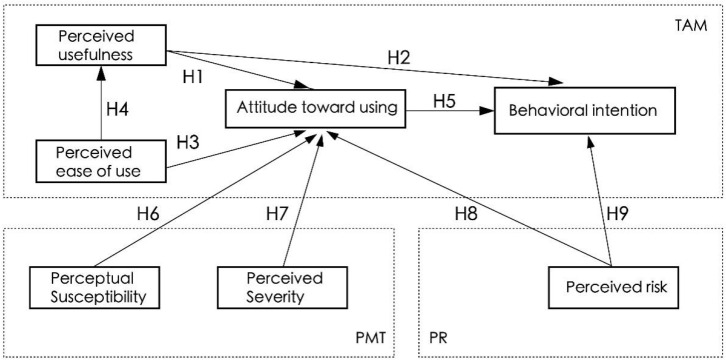
Research model.

Drawing on the previous research and integrating the TAM model, PMT, and perceived risk theory, the research model followed in this paper was formulated based on the above hypotheses, as shown in Figure 2. 

### 3.3. Variable Definition and Measurement

This study explored the views and attitudes that affect the use of mobile healthcare applications by the elderly. This study integrates the TAM, protection motivation theory, and perceived risk theory into a new research model, and identifies seven factors that ultimately affect behavioral intention, including perceived ease of use (PEOU), perceived usefulness (PU), use attitude (AT), behavioral intention (BI), perceived susceptibility (PSu), perceived severity (PSe), and perceived risk (PR). In order to ensure the reliability and validity of the variables, the design of the questionnaire items mainly drew on the measurement items commonly used in domestic and foreign literature research, and some items were modified according to the characteristics of the mobile healthcare application itself. After referring to the questionnaire and structural design of Davis (1989) and others, this study was able to specify 31 questions. Table 1 provides the definition of operability, measurement items, and reference sources of the scale.

## 4. Empirical Analysis

### 4.1. Questionnaire Design

A questionnaire survey was employed in this study for empirical research. For better statistics of the service condition of mobile health apps from the aged and valid data acquisition, the application scopes were defined as (1) hospital mobile health apps; (2) community health service apps; (3) mobile apps developed by third-party organizations; (4) smart healthcare service product health apps. In consideration of facilitating a better comprehension of the research topics for the aged, the first part of the questionnaire defined the concept of mobile healthcare application. Apps such as “Chunyu Doctor”, “Pingan Health”, and “DXY” that are most commonly used in China were listed in the questionnaire for the aged to obtain a more intuitive understanding.

This paper performed questionnaire pretesting for reliability validation prior to the formal survey. The pretesting was conducted from 5 to 23 March 2022, with 50 copies distributed and 46 valid questionnaires received. We analyzed the reliability of the pretest questionnaire and eliminated undesirable items for better option reliability.

An online questionnaire of this study was distributed to aged users of mobile health apps from April to June 2022 (this questionnaire began with a question regarding whether the respondents use mHealthcare Apps; responses with the answer “No” would be excluded as invalid). Respondents could access the questionnaire by clicking the URL link. In addition to basic personal data, degrees of agreement or disagreement of the respondents were indicated using a 7-point Likert scale, ranging from 1 (strongly disagree) to 7 (strongly agree). The aged respondents agreed to fill out the questionnaire on a fully informed and voluntary basis and were given the option to opt out at any time during the survey. A total of 455 samples were collected, of which 365 samples were valid after eliminating invalid ones (such as logical errors and repetition), with a validity rate of 80.21%. In the end, we employed the software SPSS 22.0 and AMOS17.0 (IBM Corp., Armonk, NY, USA) to analyze the collected valid data.

### 4.2. Descriptive Statistics

The population variable distribution is shown in Table 2. There were 160 male respondents, accounting for 43.84%, and 205 female respondents, accounting for 56.16%, slightly more than the males; in terms of age, the proportions of the aged who were 60–65 years old, 66–70 years old, 71–75 years old, and over 76 years were 43.01%, 29.59%, 17.81%, and 9.59%, respectively, showing that there was a greater number of users who were 60–65 years old and 66–70 years old, covering the main proportion of the respondents; in terms of educational background, the proportions of junior high school and under, senior high school, junior college, undergraduate, and Master’s and above were 48.77%, 22.47%, 13.15%, 10.41%, and 5.2%, respectively; in terms of occupation, the proportions of government departments and public institutions, private business owners or managers, professionals and technical personnel, service personnel, industrial workers, and agricultural laborers were 9.86%, 6.85%, 6.3%, 7.4%, 9.86%, and 59.73%, respectively; in terms of monthly income, the proportions of below CNY 2000, CNY 2001–3500, CNY 3501–5000, and over CNY 5000 were 15.89%, 48.22%, 21.92%, and 13.97%, respectively. Overall, the samples collected in this study are relatively equally distributed in each range of demographic variables, which can better reflect the participating subjects involved in this study.

### 4.3. Scale Reliability Analysis

Reliability refers to the consistency or stability of the measured results from the measurement index, which reflects the reliability degree of the measured feature. This study adopted Cronbach’s α of the statistical software Spss 22.0(IBM Corp., Armonk, NY, USA) for the scale reliability review. As shown in Table 3, values of Cronbach’s α were 0.903, 0.931, 0.894, 0.919, 0.923, 0.899, and 0.961 for perceived ease of use, perceived usefulness, attitude toward using, behavioral intention, perceived susceptibility, perceived severity, and perceived risk, respectively, and the total Cronbach α was 0.832, all of which are higher than 0.7, indicating good scale reliability.

### 4.4. Scale Validity Analysis

#### 4.4.1. KMO and Bartlett Tests

This study mainly used the method of factor analysis to analyze the structural validity. In order to carry out an effective analysis, the sample data of this study were divided into two parts. Half of the samples were used for exploratory factor analysis, and the other half were used for confirmatory factor analysis. This research mainly employed factor analysis to analyze the structural validity. Normally, the KMO and Bartlett tests are performed prior to factor analysis. The KMO value represents the specific value of all correlation coefficients and the net correlation coefficients related to the variable, and the greater the value is, the better the correlation will be. KMO statistics values above 0.5 are fit for factor analysis. A larger statistics number of Bartlett’s sphericity test, with a corresponding concomitant probability less than the established significance level, indicates the results reject the null hypothesis and the data under analysis are of relatively high validity and fit for factor analysis. As shown in Table 4, the KMO value of the scale was 0.9, the *p* value of Bartlett’s sphericity test was 0, and the preliminary validity test was good.

#### 4.4.2. Exploratory Factor Analysis

We used half of the sample data for exploratory factor analysis. The factor loading of each item was larger than 0.7, and the cumulative percentage of the variance explained was 84.549%, indicating a high validity of the abstracted common factors. Thus, based on Table 5, in total, seven common factors were abstracted, consistent with the pre-fractal dimensions and further indicating good validity.

### 4.5. Measurement Model

#### 4.5.1. Convergent Validity

Convergent validity measures whether the measurement indicators intensively reflect the measured factors, which is usually measured by the average variance extracted (AVE). The greater the AVE value, the better the measurement index can reflect the factor to be measured. Fornell and Larker [60] proposed that the convergent validity is good when the AVE value is greater than 0.5, and acceptable when the value is between 0.36 and 0.5. It can be seen from Table 6 that the AVE values of the constructs of the samples (half of the research samples) in this study were greater than 0.5; the standardized factor loadings for all the variables were greater than 0.7; and all the CR values were greater than 0.8, indicating the good convergent validity of the scale, with a better performance of the measurement index in reflecting the factors to be measured.

#### 4.5.2. Discriminant Validity

Discriminant validity is employed to measure the degree to which a construct differs from others. According to [60], a model measurement would have good discriminant validity if the AVE square roots are larger than the correlation coefficients between this construct and others. It can be seen from Table 7 (half of the study samples) that the square root of each construct’s AVE is greater than the correlation coefficient between this construct and all other constructs, so it can be shown that the discriminant validity of this measurement is good.

### 4.6. Structural Model Analysis

#### 4.6.1. Model Fit Criteria

We carried out model fit measurement. A good model fit indicates a close approximation between the model and the sample. Based on the research by [61,62,63,64], and other scholars, this study selected a number of indexes (MLχ^2^, DF, χ^2^/DF, RMSEA, SRMR, TLI, CFI, NFI, PGFI, PNFI, IFI) to evaluate the structural model fit. Upon the parameter measurement as per the model and hypotheses, the results, as shown in Table 8, prove that the model has a good fit, and the hypothetical theoretical framework is consistent with the results. 

#### 4.6.2. Path Analysis

As shown in Table 9, perceived usefulness (R = 0.22, *p* < 0.001) has a positive impact on the attitude of the aged toward using mobile health apps, indicating that H1 is supported. Perceived usefulness (R = 0.22, *p* < 0.001) has a positive impact on the behavioral intention of the aged to use mobile health apps, indicating that H2 is supported. Perceived ease of use (R = 0.22, *p* < 0.001) has a positive impact on the attitude of the aged toward using mobile health apps, indicating that H3 is supported. Perceived ease of use (R = 0.22, *p* < 0.001) has a positive impact on perceived usefulness, indicating that H4 is supported. Attitude toward using (R = 0.38, *p* < 0.001) has a positive impact on behavioral intention, indicating that H5 is supported. Perceived susceptibility (R = −0.06, *p* = 0.19) is not significantly related to the attitude of the aged toward using mobile health apps, indicating that H6 is not supported. Perceived severity (R = 0.35, *p* < 0.001) has a positive impact on the attitude of the aged toward using mobile health apps, indicating that H7 is supported. Perceived risk (R = −0.22, *p* < 0.001) has a negative impact on the attitude of the aged toward using mobile health apps, indicating that H8 is supported. Perceived risk (R = −0.22, *p* < 0.001) has a negative impact on the behavioral intention of the aged to use mobile health apps, indicating that H9 is supported.

### 4.7. Hypothesis Explanation

**Figure 3:**
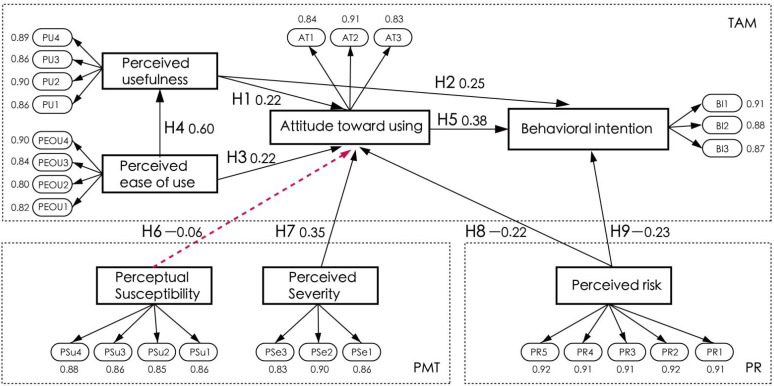
Hypothesis path coefficient diagram.

Regression coefficients of the SEM model of this study are shown in Figure 3. The larger the coefficient, the greater the role the independent variable has in the dependent variable. All the hypotheses of the model are supported, except for H6. 

## 5. Results and Discussion

Mobile healthcare applications can mainly provide the aged with medical health information consultation, health management, and medical services. They feature information technology characteristics and are concerned with health. Therefore, this paper aims to build a research model of behavioral intention to use mobile health apps from the relevant technical perspective and health behavior perspective in combination with the TAM, PMT, and perceived risk theory. We carried out a questionnaire survey and analyzed the correlation of each variable in the theoretical model via the structural equation model. The results of the empirical analysis revealed a number of significant findings, as discussed below: 

1. From the perspective of technology acceptance, our empirical research found that perceived usefulness, perceived ease of use, and behavioral attitude significantly affect the user’s behavioral willingness to use mobile health services. This is consistent with the research results of [65,66], and others. The order of influence is H4 (R = 0.60, *p* < 0.001), H5 (R = 0.38, *p* < 0.001), H2 (R = 0.22, *p* < 0.001), H1 (R = 0.22, *p* < 0.001), and H3 (R = 0.22, *p* < 0.001). The H4 path coefficient is the highest, and perceived ease of use is an important factor affecting the perceived usefulness of the elderly. This may be due to the physical and psychological impact on the elderly, who encounter numerous challenges with the use of information technology [67]. When the elderly feel that the mobile healthcare application is easy to use, they will also feel that it is useful. The establishment of H3 also shows that perceived ease of use significantly affects behavior and attitude. Therefore, the simpler and more convenient the mobile healthcare application is, the more useful the mobile healthcare application will be for the elderly. Therefore, when we design a mobile healthcare application for the elderly, we must consider the ease of use of the application and weaken the technical barriers for the elderly to use the mobile health service [68]. The second is the H5 path coefficient, which indicates that the elderly will have a greater behavioral intention when they have a good attitude toward mobile healthcare applications. In recent years, affected by COVID-19, older people have gradually realized that mHealthcare can change their own behavior and lifestyle [69]. This change in attitude will also encourage older people to adopt behavioral intentions. Next is the H2 path coefficient. Perceived usefulness is an important factor that affects the behavioral intention of the elderly, which indicates that when the elderly feel that mobile healthcare applications are useful, they will develop a behavioral intention to use them. The establishment of H1 also shows that perceived usefulness is an important factor affecting the behavioral intention of the elderly. This means that when the elderly feel that mobile healthcare applications can meet the needs of elderly care and health, they will have a positive attitude; therefore, application service providers should improve the “usefulness” of mobile healthcare applications, provide targeted services for the elderly, and make the apps feel useful.

2. From the perspective of health behavior, H7 is established, and there is a positive correlation between perceived severity and use attitude, which is consistent with the research results of [55,70,71], and others. This shows that the elderly will have a greater chance of using mobile healthcare applications when they feel that their health is seriously threatened. The reason may be that when the elderly feel that their health is seriously threatened, they will try to obtain more resources to maintain their health, and the services provided by mobile healthcare applications can help the elderly to improve their health. This also requires application service providers to provide more targeted services to make the elderly feel that apps are useful when their own health is seriously threatened, so as to encourage the adoption of mobile healthcare applications.

Many previous empirical studies using PMT have presented different conclusions on the effects of the two variables of threat assessment (threat susceptibility and threat severity) and behavioral willingness in different scenarios. For example, in a study of information security, Zhang and McDowell [72] found that users’ threat assessment of information security has no significant relationship with their willingness to use strong passwords; while, in a study of Internet plagiarism, Lee [73] found that users’ threat assessment has a positive impact on their willingness to use anti-plagiarism software. This study empirically proves that H6 is untenable, which indicates that there is no significant relationship between perceived susceptibility and attitude to use, which is inconsistent with the results of empirical studies such as [49,50,51]. This may be due to the lack of mobile healthcare application knowledge and serious information asymmetry among the elderly in China [30]. Failure to connect their own health with mobile healthcare applications leads to a lack of significant impact of perceived susceptibility on the attitude toward using mobile healthcare applications. This requires expanding the spread of mobile healthcare applications among the elderly, so that the elderly can understand mobile healthcare applications and establish effective links.

3. From the perspective of perceived risk, through empirical findings, we determined that perceived risk is a negative correlation factor that significantly affects the attitude and willingness of the elderly to use mobile healthcare applications. This is consistent with the findings of [32,33,34,53,55]. When the elderly feel that there are risks in using mobile health apps and hidden risks in information security, they will be less willing to use such apps [38]. Therefore, it is necessary to reduce the use risk concerns of the elderly, such as improving the privacy protection and information security level of mobile healthcare applications and improving the service quality of health information.

## 6. Suggestions and Conclusions

### 6.1. Suggestions

This research is aimed to promote the behavioral intention of the aged to adopt mHealthcare apps, and to provide suggestions for improving the overall service quality and sustainable development of mHealthcare apps. According to the empirical conclusions, this study has proposed suggestions from the perspectives of technology acceptance, health behavior, and perceived risk, including: (1) promote the usefulness and ease of use of mHealthcare apps; (2) strengthen the efforts on the publicity of mHealthcare apps; (3) reduce the risks of using mHealthcare apps.

#### 6.1.1. Promote the Usefulness and Ease of Use of mHealthcare Apps

The research has proven that perceived usefulness has a positive influence on the attitude of the aged toward using mHealthcare apps. As for the aged, an accurate grasp of mHealthcare service needs is an important approach for behavioral intention enhancement, and also an important basis for the rendering of aged-care service by the concerned mHealthcare service providers and departments [40]. MHealthcare service providers should strengthen the service awareness and proceed in all cases from the “needs of customers” [74] to render dedicated services for the aged to achieve perceived usefulness: for example, arranging specific explications of common chronic diseases among the aged in the health knowledge module targeted for the aged, and inviting doctors to regular online live streaming, where the aged user can achieve direct online communication with the doctors. 

MHealthcare service providers should become acquainted with the real demands of the aged via means such as observations and visits; they should classify and summarize the survey results, improve the service content and modes, have an accurate command of the mHealthcare service demands from the aged, and provide targeted service based on different levels and needs.

As indicated in this research, perceived ease of use has a positive impact on the attitude of the aged toward using mHealthcare apps. As a consequence, the perceived ease of use of mHealthcare apps needs to be promoted. Firstly, most of the aged physiologically feature a gradual decline in memory ability, learning ability, visual and hearing ability, and operation ability. Psychologically, the aged regard mHealthcare apps as the creatures of high technology, which require a brand-new, huge, and complex knowledge system for use and operation, consequently forming barriers in the acceptance and usability of mHealthcare apps [75]. Conformance to the physiological and psychological features of the aged [76], as well as their use habits, should be achieved during the development of mHealthcare apps. For one thing, unnecessary operation steps should be reduced as much as possible; for another, reasonable guidance, such as information tips for operation manners and objectives, should be timely added in the steps where comprehension impediments of the aged can easily occur, as well as rewards for operation feedback or certain task fulfillment. Such measures would not only facilitate the acceptance of the operation purpose by the aged, but also effectively enhance the operating efficiency thereof [77].

Relevant practical technical training should be provided to the aged to enhance their willingness to use mHealthcare apps. Lastly, the influence from the relation among relatives and friends should also be highly valued. Those who have a significant impact on the aged can effectively eliminate the psychological resistance of the aged to mHealthcare apps and can also teach the aged in detailed operation. 

#### 6.1.2. Strengthen the Efforts on the Publicity of mHealthcare Apps

According to the research, perceived susceptibility is not significantly related to the attitude of the aged toward using mHealthcare applications. This indicates that the aged have not yet effectively connected their own health issues with the use of mHealthcare apps, which assigns the following tasks to mHealthcare application developers: to deliver targeted services based on the characteristics of the aged, formulate strong goal-oriented marketing programs to serve the aged with satisfactory mHealthcare application service, establish effective links, strengthen the efforts on the publicity of mHealthcare apps, timely provide the aged with abundant education activities concerning mHealthcare apps, create a sound operation atmosphere, and build and maintain long-term relationships with the aged. mHealthcare app advocacy can be arranged for the aged by the government, media, and health education institutions. In the meantime, medical institutions and authoritative medical experts can guide and recommend the aged to use effective mHealthcare apps.

#### 6.1.3. Reduce the Risks of Using mHealthcare Apps

We can see from the research that perceived risk has a negative impact on the attitude of the aged toward using and their behavioral intention. The aged may frequently encounter problems such as false advertisement, online rumors, and Internet fraud while surfing the net, giving rise to demands to solve the concerns of the aged about the risk of use, information security, and other issues that may occur during their use of mHealthcare apps. We need to mitigate their concerns about the risk of use.

As a significant carrier of health services, it is of great importance to upgrade the privacy protection and information security level of mHealthcare apps, and the government should establish an mHealthcare-app-targeted information security assurance system, form a security supervision mechanism with judicial and public security departments, jointly solve network information security problems, standardize the health information market, and crack down on online rumors, Internet fraud, and other issues. Government departments should focus more on the quality assessment of health information and formulate relevant regulations to punish websites, applications, or individuals that maliciously disseminate false information [78].

### 6.2. Conclusions

This study, which is different from the previous one, focuses on China’s aged group who have a strong demand for medical health. Consequently, this study employs new perspectives and particularly considers the impact of health factors on the use intention of mHealthcare apps. This study incorporates the protection motivation theory-based perceived severity and perceived susceptibility into the model, probes into the use intention of the aged toward mHealthcare apps, and combines the technology level (perceived usefulness, perceived ease of use, attitude toward using, and behavioral intention) and the perceived risk. PMT was previously more commonly used in the field of healthcare. Our adoption of PMT in the use intention of the aged toward mHealthcare apps enriches its application scenarios and is of great theoretical research significance.

Through empirical research and analysis, this study has obtained some valuable conclusions: from the perspective of technology acceptance, perceived usefulness and perceived ease of use significantly affect the attitude of the elderly toward mHealthcare apps; perceived usefulness and use attitude positively affect use intention; perceived ease of use has a positive impact on perceived usefulness. From the perspective of health, perceived severity has a significant positive correlation with the use attitude of the elderly; perceived susceptibility has no significant impact on the attitude of the elderly to use mHealthcare apps. From the perspective of perceived risk, perceived risk has a significant negative correlation with the use attitude and behavior intention of the elderly.

This study can help people understand the actual needs of the elderly for healthy mobile applications and the role of these applications. In terms of the practical value of this study, it provides a reference for the research of user behavior of other similar healthy mobile applications, and provides targeted suggestions for the development of healthy mobile applications for the elderly. Meanwhile, it has important practical significance to improve the overall service quality of mHealthcare apps, the level of mHealthcare apps, and the health literacy of the elderly.

## 7. Limitations and Future Research

1. Although all the conformations in this study’s model are related, there may be some potential variables or second-order dimensions that have not been studied. Therefore, future researchers can add new dimensions, including sub-dimensions and intermediary variables, to improve the model by strengthening its explanatory power.

2. This study has no in-depth or specific discussion on the functional differences of mobile healthcare applications. In the future, we can have a more in-depth discussion based on the functional differences of mobile healthcare applications.

3. This study only conducted a questionnaire survey for the elderly but did not conduct a more in-depth study based on the health characteristics of the population. Therefore, researchers can further study the differences between the elderly with different health levels to produce more specific results and countermeasures for different groups.

4. This study takes structural equation modeling as the main research and analysis method for quantitative research. In the future, qualitative research (expert interviews and field work) can be added to supplement the deeper meaning that quantitative data cannot express.

## Figures and Tables

**Table 1:** Variables and indicators of the research.

Category	Research Variables	Definition of Operability	Code	Measurement Item	Sources
TAM	Perceived ease of use (PEOU)	The easier the aged find the use of mHealthcare apps, the more likely they are to have a positive attitude toward using them	PEOU1	1. MHealthcarecare Apps can upgrade our health quality	[17,56,57]
			PEOU2	2. I think mHealthcare Apps have made my daily life safer	
			PEOU3	3. MHealthcare services have enriched my access for disease prevention and treatment	
			PEOU4	4. I think mHealthcare Apps are useful	
	Perceived usefulness (PU)	When the aged feel helped during the use of mHealthcare apps, they will enhance their perceived usefulness toward the apps, thus forming a positive attitude toward using them	PU1	1. I think it’s easy to use mHealthcare Apps	[17,56,57]
			PU2	2. I thinks it’s easy to learn how to operate the mHealthcare Apps	
			PU3	3. I think the mHealthcare Apps are simple and easy to use	
			PU4	4. In general, mHealthcare Apps are easy to use	
	Attitude toward using (AT)	The more active the aged are during the use of mHealthcare apps, the more likely they will be to access the platform	AT1	1. It’s a good idea to use mHealthcare service for health management	[17,56,57]
			AT2	2. I think my health status can be improved by using mHealthcare services	
			AT3	3. I think mHealthcare services are very valuable	
	Behavioral intention (BI)	The more positive the attitude of the aged toward using mHealthcare apps, the more positive the behavior trend will be, which will lead to the use of such apps	BI1	1. I’d love to use mHealthcare services for health management	[17,56,57]
			BI2	2. I’m planning to learn how to use mHealthcare services	
			BI3	3. I prefer mHealthcare services to other forms of health management	
PMT	Perceived susceptibility (PSu)	Individual judgment of probability of occurrence of threat events from which they may suffer	PSu1	1. I think I’m more susceptible to illness than other people	[24,57]
			PSu2	2. I feel that I am likely to have chronic diseases such as high blood pressure/heart disease/diabetes in the future	
			PSu3	3. I find my physical condition is getting worse	
			PSu4	4. I find myself in a state of sub-health	
	Perceived severity (PSe)	Severity of threat events’ consequences or degree of harmfulness to them upon individual judgment	PSe1	1. I think the chronic diseases in elderly such as high blood pressure and heart disease may endanger my life	[24,57]
			PSe2	2. I think the deficient knowledge about the aged care may cause me to miss the optimal treatment	
			PSe3	3. I think my life and work will be disturbed by any disease	
Perceived risk	Perceived risk (PR)	The aged perceive the use of mobile health apps as risky	PR1	1. I think the adoption of mHealthcare services may lead to privacy disclosure	[55,58,59]
			PR2	2. I think the adoption of mHealthcare services may fail to meet my original expectation	
			PR3	3. There may be security issues such as function disorders/system breakdown during the use of mHealthcare services	
			PR4	4. Adoption of mHealthcare services may lead to financial losses, such as additional unknown paid services in the service system	
			PR5	5. Adoption of mHealthcare services makes me nervous or anxious	

**Table 2:** Basic information of respondents.

Frequency Analysis Results				
Item	Option	Frequency	Percentage (%)	Cumulative Percentage (%)
Gender	Male	160	43.84	43.84
	Female	205	56.16	100.00
Age	60–65 years old	157	43.01	43.01
	66–70 years old	108	29.59	72.60
	71–75 years old	65	17.81	90.41
	Over 76 years old	35	9.59	100.00
Educational background	Junior high school and under	178	48.77	48.77
	Senior high school	82	22.47	71.24
	Junior college	48	13.15	84.39
	Undergraduate	38	10.41	94.80
	Master’s and above	19	5.20	100.00
Occupation	Government departments and public institutions	36	9.86	9.86
	Private business owners or managers	25	6.85	16.71
	Professionals and technical personnel	23	6.30	23.01
	Service personnel	27	7.40	30.41
	Industrial workers	36	9.86	40.27
	Agricultural laborers	218	59.73	100.00
Monthly income	Below CNY 2000	58	15.89	15.89
	CNY 2001–3500	176	48.22	64.11
	CNY 3501–5000	80	21.92	86.03
	Over CNY 5000	51	13.97	100.00
Total		365	100.00	100.00

**Table 3:** Cronbach reliability analysis.

Variables	Item	Corrected Item-Total Correlation (CITC)	Cronbach’s α if Item Deleted	Cronbach’s α	Total Cronbach α
PEOU	PEOU1	0.756	0.885	0.903	0.832
	PEOU2	0.750	0.887		
	PEOU3	0.769	0.880		
	PEOU4	0.860	0.847		
PU	PU1	0.821	0.916	0.931	
	PU2	0.858	0.903		
	PU3	0.828	0.913		
	PU4	0.845	0.907		
AT	AT1	0.778	0.861	0.894	
	AT2	0.827	0.818		
	AT3	0.772	0.866		
BI	BI1	0.855	0.867	0.919	
	BI2	0.833	0.885		
	BI3	0.818	0.897		
PSu	PSu1	0.819	0.901	0.923	
	PSu2	0.817	0.902		
	PSu3	0.820	0.901		
	PSu4	0.833	0.897		
PSe	PSe1	0.794	0.861	0.899	
	PSe2	0.831	0.829		
	PSe3	0.776	0.876		
PR	PR1	0.884	0.952	0.961	
	PR2	0.892	0.951		
	PR3	0.888	0.952		
	PR4	0.884	0.952		
	PR5	0.897	0.950		

**Table 4:** KMO and Bartlett tests.

KMO Value		0.9
Bartlett’s Test of Sphericity	Chi-square Approximation	4695.093
	df	325
	*p* value	0

**Table 5:** Factor loadings (rotated).

Name	Factor Loading							Commonality (Common Factor Variance)
	Factor 1	Factor 2	Factor 3	Factor 4	Factor 5	Factor 6	Factor 7	
PEOU1	−0.115	0.239	0.019	**0.800**	0.138	0.202	0.099	0.780
PEOU2	−0.122	0.222	0.078	**0.763**	0.103	0.173	0.114	0.706
PEOU3	−0.110	0.208	0.026	**0.776**	0.128	0.075	0.268	0.752
PEOU4	−0.074	0.172	0.048	**0.882**	0.153	0.127	0.134	0.873
PU1	−0.096	**0.855**	0.010	0.146	0.174	0.173	0.157	0.847
PU2	−0.106	**0.857**	−0.012	0.249	0.172	0.177	0.107	0.880
PU3	−0.145	**0.848**	0.073	0.220	0.095	0.147	0.189	0.860
PU4	−0.067	**0.865**	0.000	0.251	0.101	0.058	0.183	0.862
AT1	−0.233	0.253	0.047	0.207	0.245	0.204	**0.742**	0.816
AT2	−0.269	0.247	0.035	0.248	0.215	0.172	**0.778**	0.877
AT3	−0.150	0.243	−0.031	0.254	0.209	0.206	**0.785**	0.850
BI1	−0.262	0.175	−0.042	0.176	0.241	**0.816**	0.204	0.897
BI2	−0.240	0.150	−0.040	0.218	0.170	**0.824**	0.172	0.866
BI3	−0.213	0.249	−0.011	0.221	0.172	**0.810**	0.153	0.866
PSU1	0.042	0.068	**0.895**	0.006	0.049	−0.050	−0.009	0.812
PSU2	−0.020	0.019	**0.903**	0.022	0.098	−0.046	−0.027	0.829
PSU3	0.062	−0.027	**0.895**	0.037	0.046	0.037	0.069	0.816
PSU4	0.043	−0.003	**0.919**	0.074	0.013	−0.002	0.005	0.853
PSE1	−0.099	0.158	0.078	0.170	**0.832**	0.134	0.265	0.851
PSE2	−0.074	0.111	0.087	0.168	**0.878**	0.160	0.165	0.877
PSE3	−0.139	0.226	0.079	0.146	**0.827**	0.206	0.101	0.835
PR1	**0.899**	−0.095	0.038	−0.068	−0.068	−0.175	−0.072	0.863
PR2	**0.914**	−0.044	0.017	−0.061	−0.100	−0.140	−0.114	0.884
PR3	**0.915**	−0.082	0.046	−0.112	−0.058	−0.080	−0.104	0.880
PR4	**0.908**	−0.085	0.012	−0.100	−0.094	−0.113	−0.120	0.877
PR5	**0.899**	−0.116	0.039	−0.098	−0.035	−0.147	−0.136	0.875
Cumulative percentage of variance explained %								84.549

Note: Bold numbers mean that factor loading is greater than 0.5.

**Table 6:** Summary table of confirmatory factor analysis.

Item			Estimate	S.E.	C.R.	*p*	Std	CR	AVE
PEOU1	<---	PEOU	1				0.779	0.910	0.718
PEOU2	<---	PEOU	1.065	0.090	11.889	***	0.839		
PEOU3	<---	PEOU	1.116	0.089	12.489	***	0.872		
PEOU4	<---	PEOU	1.142	0.089	12.851	***	0.894		
PU1	<---	PU	1				0.829	0.915	0.728
PU2	<---	PU	1.032	0.074	13.898	***	0.876		
PU3	<---	PU	0.919	0.072	12.695	***	0.824		
PU4	<---	PU	1	0.071	14.078	***	0.883		
AT1	<---	AT	1				0.816	0.875	0.702
AT2	<---	AT	1.187	0.091	13.104	***	0.906		
AT3	<---	AT	0.964	0.085	11.312	***	0.786		
BI1	<---	BI	1				0.892	0.909	0.769
BI2	<---	BI	0.943	0.060	15.809	***	0.886		
BI3	<---	BI	0.980	0.066	14.812	***	0.852		
PSu1	<---	PSu	1				0.866	0.919	0.739
PSu2	<---	PSu	1.026	0.073	14.082	***	0.846		
PSu3	<---	PSu	1.009	0.068	14.894	***	0.875		
PSu4	<---	PSu	1.008	0.071	14.242	***	0.852		
PSe1	<---	PSe	1				0.831	0.887	0.724
PSe2	<---	PSe	1.029	0.075	13.654	***	0.897		
PSe3	<---	PSe	0.944	0.076	12.357	***	0.822		
PR1	<---	PR	1				0.906	0.958	0.819
PR2	<---	PR	0.987	0.052	19.051	***	0.906		
PR3	<---	PR	1.009	0.054	18.793	***	0.902		
PR4	<---	PR	0.951	0.052	18.168	***	0.890		
PR5	<---	PR	1.041	0.052	19.875	***	0.921		

Note: *** *p* < 0.001.

**Table 7:** Discriminant validity of the measurement model.

	AVE	PEOU	PU	AT	BI	PSu	PSe	PR
PEOU	0.718	**0.847**						
PU	0.728	0.636 ***	**0.853**					
AT	0.702	0.546 ***	0.504 ***	**0.838**				
BI	0.769	0.539 ***	0.555 ***	0.596 ***	**0.877**			
PSu	0.739	0.203 *	0.037	0.037	0.053	**0** **.860**		
PSe	0.724	0.592 ***	0.520 ***	0.616 ***	0.596 ***	0.197*	**0** **.851**	
PR	0.819	−0.333 ***	−0.260 **	−0.428 ***	−0.431 ***	−0.079	−0.333 ***	**0** **.905**

Note: The square root of AVE (shown as bold at diagonal). * *p* < 0.05 ** *p* < 0.01 *** *p* < 0.001.

**Table 8:** Model fit index.

Model Fit	Criteria	Model Fit of Research Model	Judgment
ML chi-square (MLχ^2^)	The smaller, the better	345.801	
Degrees of freedom (DF)	The larger, the better	278	
Normed chi-square χ^2^/df	<3	1.244	Yes
Root mean square error of approximation (RMSEA)	<0.08	0.038	Yes
Standardized root mean square residual (SRMR)	<0.08	0.0434	Yes
Tucker–Lewis index (TLI)	>0.9	0.978	Yes
Comparative fit index (CFI)	>0.9	0.981	Yes
Normative fit index (NFI)	>0.9	0.912	Yes
Parsimony goodness-of-fit index (PGFI)	>0.5	0.690	Yes
Parsimony normed fit index (PNFI)	>0.5	0.780	Yes
Incremental fit index (IFI)	>0.9	0.981	Yes

**Table 9:** Path analysis.

Hypothesis	Route			Estimate	S.E.	C.R.	*p*	STD	Results
H1	AT	<---	PU	0.184	0.047	3.882	***	0.217	Support
H2	BI	<---	PU	0.245	0.052	4.703	***	0.249	Support
H3	AT	<---	PEOU	0.203	0.061	3.315	***	0.217	Support
H4	PU	<---	PEOU	0.664	0.06	10.984	***	0.603	Support
H5	BI	<---	AT	0.443	0.07	6.34	***	0.383	Support
H6	AT	<---	PSu	−0.049	0.037	−1.311	0.19	−0.057	Nonsupport
H7	AT	<---	PSe	0.308	0.049	6.239	***	0.346	Support
H8	AT	<---	PR	−0.157	0.032	−4.89	***	−0.223	Support
H9	BI	<---	PR	−0.184	0.041	−4.551	***	−0.225	Support

Note: *** *p* < 0.001.

## Data Availability

Not applicable.
